# Cardiovascular complications of pediatric chronic kidney disease

**DOI:** 10.1007/s00467-006-0359-0

**Published:** 2008-01-01

**Authors:** Mark M. Mitsnefes

**Affiliations:** 1 Division of Nephrology and Hypertension, Cincinnati Children’s Hospital Medical Center, Cincinnati, OH USA; 2 Division of Nephrology and Hypertension, MLC: 7022, 3333 Burnet Avenue, Cincinnati, OH 45229-3039 USA

**Keywords:** Chronic kidney disease, Children, Cardiovascular disease, ESRD

## Abstract

Cardiovascular disease (CVD) mortality is a leading cause of death in adult chronic kidney disease (CKD), with exceptionally high rates in young adults, according to the Task Force on Cardiovascular Disease. Recent data indicate that cardiovascular complications are already present in children with CKD. This review summarizes the current literature on cardiac risk factors, mortality and morbidity in children with CKD.

**Learning objectives:**

1. To review recent data on the epidemiology of CVD in children with CKD
2. To understand the mechanisms of cardiovascular abnormalities in pediatric CKD
3. To review recent advances in the diagnosis and clinical presentation of cardiovascular complications in children with CKD
4. To outline current understanding in the strategies to prevent progression of CVD in children with CKD.

## Cardiovascular mortality and morbidity

The survival of children with CKD in the U.S. remains low: for children on dialysis the lifespan is 40–60 years less and for transplant patients, about 20–25 years less than that of an age- and- race-matched US population [1, 2]. The most likely cause of this is increased cardiovascular mortality due to the development of accelerated ischemic heart disease and premature dilated cardiomyopathy. The evidence comes from studies of young adults who developed renal failure during childhood. Oh et al. [3] analyzed the outcome of 283 young adults with childhood onset CKD between 1970 and 1997. Fifty percent of the deceased patients had died of cardiovascular or cerebrovascular events. Groothoff et al. [4] conducted a national retrospective and prospective cross-sectional study to evaluate the late physical, social and psychological effects of renal insufficiency (LERIC) in all Dutch children who started renal replacement therapy between 1972 and 1992. Of 381 patients, 85 had died. Cardiovascular disease was the most common cause of death and accounted for 41% of all deaths. Cerebrovascular accident, congestive heart failure, myocardial infarction and cardiac arrest (respectively) were the most common causes of cardiac death. Similar analysis of long-term survival from the Australia and New Zealand Dialysis and Transplant Registry [5] of all children and adolescents who were under 20 years of age when renal replacement therapy commenced (study period was 40 years) showed mortality rates 30 times higher than in the age-matched general population; CVD was the most common cause of death (45%).

Cardiovascular death happens not only in later life, but also in childhood CKD. In the general pediatric population, the incidence of annual death due to cardiac disease is less than 3%. Yet annual reports from the United States Renal Data System (USRDS) indicate that over the last decade CVD has remained the second most common cause of death in children on chronic dialysis or after transplantation, accounting for approximately 20–25% of all deaths. Parekh et al. [6], using the USRDS database, performed a detailed cross-sectional analysis to evaluate the risk of a cardiac death in children and young adults (age 0–30 years) and to identify factors potentially associated with CVD mortality. A total of 1,380 deaths between 1990 and 1996 were analyzed. There were 311 cardiac deaths (22.5% of the total). Cardiac deaths in children and young adults in whom ESRD developed during childhood were approximately 1,000 times more frequent than in the general pediatric population. Of the specific categories of cardiovascular deaths, cardiac arrest was the most common cause in each of the age groups, followed by arrhythmia and cardiomyopathy. These causes of cardiac death are different from those of adults. In adults, coronary artery disease and chronic congestive heart failure are the leading causes of CVD mortality and, as shown by Parekh et al. [6], these causes are extremely rare in children and adults younger than 30 years of age. The incidence of cardiac arrest in the youngest age group (0–4 years) was five to ten times higher than in other age groups, perhaps, as noted by the authors, a reflection of the difficulty of ascertaining the true cause of death in young children. Some of these young children might have died from other co-morbid conditions such as congenital disorders that are not included in the USRDS database.

The high rate of sudden death in children, especially in infants with ESRD, is poorly understood and warrants further investigation. In adults, sudden death is often a result of fatal arrhythmias due to acute ischemia of preexisting atherosclerotic disease. It is believed that arrhythmias are also the likely cause of most cases of sudden cardiac death (SCD) in children. However, the origin of acquired malignant arrhythmias in children is unlikely to be an atherosclerotic lesion. Dilated, especially hypertrophic, cardiomyopathies are a leading cause of SCD in children [7]. The macroscopic and microscopic structural abnormalities in cardiomyopathies involve fibrosis and cellular hypertrophy prone to produce an electrical instability with resultant arrhythmias. Ischemia of small coronary vessel disease secondary to medial hypertrophy might result in dispersion of repolarization properties and arrhythmia from re-entrant or autonomic mechanisms. As we discuss in more detail below in this review, children with CKD develop left ventricular hypertrophy (LVH), which is frequently severe, especially in children on prolonged dialysis therapy [8, 9]. It is currently unknown if LVH in young patients with CKD is characterized by structural abnormalities similar to familial or idiopathic hypertrophic cardiomyopathies associated with SCD. Whether LVH of children with ESRD can contribute to increased SCD is also not known. Another possibility for deadly arrhythmias in children with ESRD is acute changes in the cardiac extra- or intracellular ionic milieu, especially involving abnormalities of sodium- and potassium-based repolarization currents.

Because the causes of cardiac death in children and adults are different, it is not surprising that none of the traditional or uremia-related risk factors for adult atherosclerotic CVD predicted cardiac death in the study by Parekh [6]. One of the examples presented in this paper is the effect of race on CVD death. In adults with ESRD, white males are at higher risk for CVD mortality. In contrast, black children as shown by Parekh et al. [6] appear to have an increased risk for cardiac death. Another important observation of this study is that transplant recipients had 78% lower risk of cardiac death than dialysis patients. However, the authors pointed out that the CVD mortality rate in transplanted patients was still significantly higher (approximately ten times) than in the general pediatric population.

The analysis of cardiac morbidity in children on chronic dialysis performed by Chavers et al. [10] has confirmed that cardiac disease in children is different from adults. A total of 1,454 Medicare incident pediatric (0–19 years) dialysis patients were identified from 1991 to 1996. Among them, 452 (31.2%) developed a cardiac-related event. Arrhythmia was most common (19.6%), followed by valvular disease (11.7%), cardiomyopathy (9.6%) and cardiac arrest (3%). Ischemic heart disease was extremely rare in these children.

## Risk factors for cardiovascular disease

The risk factors and pathogenic mechanisms of development of CVD in young adults who had onset of CKD in childhood are better understood than are those producing cardiac morbidity and mortality in children. The conventional thinking is that two groups of risk factors are responsible for accelerated CVD in adults with CKD (Table 1).

**Table 1:** Cardiovascular risk factors in chronic kidney disease in adults

Traditional	CKD-related
Older age	Decreased GFR
White race	Proteinuria
Male gender	Peripheral renin-angiotensin-aldosterone activity
Hypertension	Abnormal calcium and phosphorus
↑ LDL Cholesterol	Dyslipidemia
↓ HDL Cholesterol	Hypoalbuminemia
Diabetes mellitus	Hemodynamic overload
Tobacco use	Anemia
Physical inactivity	Thrombogenic factors
Psychosocial stress	Hyperhomocysteinemia
Family history of CVD	Oxidative stress
LVH	Infection (*Chlamydia pneumoniae*)
Obesity	Chronic inflammation

First, as compared to the non-uremic population, there is an over-representation in uremic patients of classical risk factors, e.g., diabetes, hypertension and hyperlipidemia. A majority of the adults who develop ESRD do so as a complication of diabetes or generalized atherosclerosis. Often cardiac disease antedates the onset of CKD in these patients. Unfortunately, children with CKD share with adults a similar high prevalence of risk factors for adult-type atherosclerotic CVD. It is also troubling that the frequencies of these traditional risk factors have not changed over last decade. The North American Pediatric Renal Trials Collaborative Studies (NAPRTCS) data demonstrate that hypertension develops at early stages of CKD (48%) and persists (50–75%) in uremic children [11–14]. Dyslipidemia is found in 70 to 90% during chronic dialysis [15–18]. Successful renal transplantation leads to a dramatic improvement in renal function and elimination of many risk factors for atherosclerotic CVD that were present while on dialysis. However, transplant recipients are not free from multiple complications, and transplantation may amplify some of the traditional risk factors. Indeed, the prevalence of hypertension in pediatric renal allograft recipients is between 50–80% [19–21]. Also, hyperlipidemia may not disappear after renal transplantation; the reported prevalence is above 50% [22–24]. The NAPRTCS data also show that the rate of obesity, another CV risk, is increasing in children with ESRD at the time of transplantation (12.4% after 1995 vs. 8% prior to 1995) [25]. Single-center study data indicate that the number of obese patients can double at 1 year after transplantation [26].

Second, there is a multitude of uremia-related risk factors for atherosclerotic CVD. In adults with CKD, an increased homocysteine level appeared to be an independent risk factor for CVD morbidity and mortality [27–29]. Anemia has been linked to negative CVD outcome [30]. Hyperphosphatemia with increased calcium-phosphorus product constitutes a risk for cardiovascular calcification, cardiac ischemia and adverse cardiovascular outcomes [31, 32]. Elevated serum C-reactive protein (CRP), a marker of systemic inflammation, has been found to be a strong predictor of cardiac morbidity and cardiac death in CKD patients [33]. Another marker of inflammation, IL-6, has been associated with increased cardiac morbidity in adults with CKD [34, 35]. The role of systemic inflammation in CKD has been reviewed recently elsewhere [36]. Inflammation is directly linked to oxidative stress, which is now considered as a hallmark of uremia [37]. Asymmetric dimethylarginine (ADMA), an endogenous inhibitor of nitric oxide synthase and a marker of oxidative stress, has been extensively evaluated in patients with CKD by Zoccali et al. [38]. The authors demonstrated that elevated ADMA per se was responsible for a 52% higher risk of death and for a 34% higher risk of cardiovascular events in dialysis patients [39]. Another subset of novel CVD risk factors is adipokines. Adipokines, leptin and adiponectin, are the products of adipose tissue involving in regulation of lipid and glucose metabolism. Abnormal adipokines are strongly linked to insulin resistance, a known CV risk in CKD [40]. It has been determined that lower plasma adiponectin concentration independently predicts increased CVD morbidity and mortality in adults with ESRD [41]. Clinical Practice Guidelines for Cardiovascular Disease in Dialysis Patients extensively reviewed the current literature on CVD biomarkers, and the reader is referred to this publication for more information [42]. As in adults, in children with CKD many of these risk factors are very prevalent (Table 2).

**Table 2:** Prevalence of risk factors for CVD in children with CKD

Risk factors	CRI^a^ (%)	Dialysis^b^ (%)	Transplant^c^ (%)	References
Hypertension	48	52–75	63–81	11^a^, 12–14^b^, 19–21^c^
Dyslipidemia	25–53	33–87	55–84	15^a^, 15–18^a, b^, 22–24^c^
Anemia	48	40–67	32–64	43^a^, 44–47^b^, 48–51^c^,
Hyperparathyroidism	32.6–43.7	58	–	43
Hyperhomocysteinemia		87–92	25–100	52^c^, 53^b, c^, 54^c^, 55^b^
↑ CRP		76	16	56^b^,57^c^
Hypoalbuminemia	–	40–60	–	43^b^, 44^b^, 58–59^b^

Recent studies in adults on chronic hemodialysis place a malnutrition-inflammation complex at the center of a debate about the role of traditional and non-traditional risk factors for poor cardiovascular outcome. This issue emerged after publication of a series of articles, summarized in the review by Nurmohamed and Nube [60], describing the phenomenon of “reverse epidemiology.” The studies have shown that in adults on chronic hemodialysis low blood pressure, low body mass index (BMI), low serum cholesterol and low serum homocysteine are often correlated with an unfavorable clinical outcome. Thus, whereas traditional risk factors of CVD are correlated with an unfavorable outcome in the general population and patients with CKD not yet on dialysis, in hemodialyzed patients, mild hypertension, hypercholesterolemia and being overweight appear to be protective and associated with an improved survival. It has been speculated that a malnutrition-inflammation-atherosclerosis complex underlies, at least partly, the phenomenon of reverse epidemiology, since malnutrition causes a low BMI, hypocholesterolemia and low serum homocysteine levels.

## Mechanisms of cardiovascular disease in chronic kidney disease

**Fig. 1:**
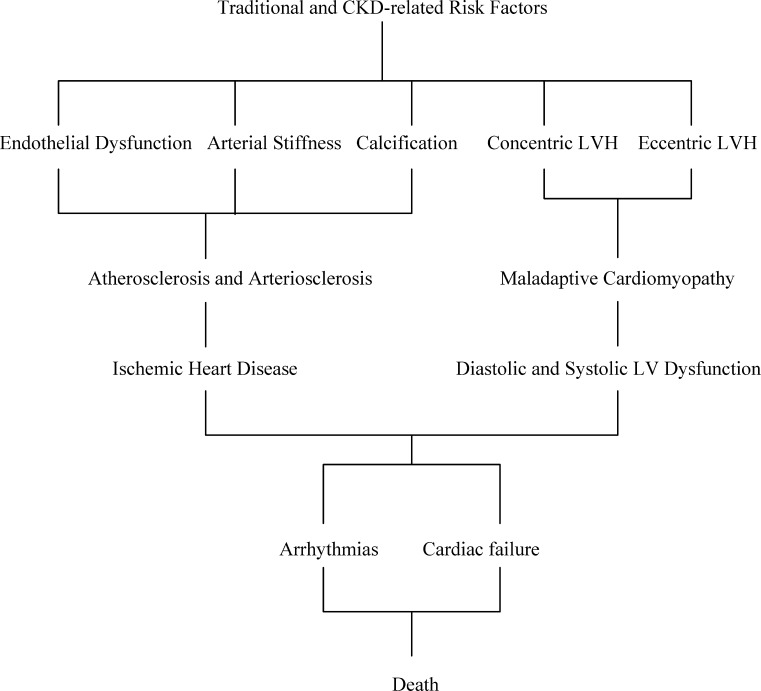
Cardiovascular disease in chronic kidney disease

There are two parallel processes involved in the development of CVD in CKD patients (Fig. 1).

The first is cardiac remodeling leading to hypertrophy of the left ventricle (LV) as a response to mechanical or hemodynamic overload. Two different patterns of LV remodeling can produce increase in LV mass (LVM) [61]. The concentric LV remodeling and hypertrophy may be the results of pressure overload as occurs with hypertension, whereas eccentric LVH may be related to volume and sodium retention, anemia and arteriovenous shunt. The patterns of sarcomere formation induced by pressure or volume overload are distinct. Pressure-induced concentric LVH is characterized by a parallel addition of sarcomeres resulting in the increase of cross-sectional area and diameter of the myocytes. Increase in LVM in this case is obtained by a marked increase in wall thickness with a less evident increase in the LV cavity that yields an elevated relative wall thickness and concentric LVH. From the physiological view, increased systolic blood pressure (BP) and pulse pressure, due to increased peripheral resistance and arterial stiffness, are the principal factors opposing LV ejection and leading to an increased LV workload and concentric LVH. An increase in LVM can also be obtained by an increase in the LV cavity with a symmetric increase in wall thickness to maintain the ratio between the wall thickness and LV transversal radius (relative wall thickness) normal, producing eccentric LVH. In this case, the addition of sarcomeres occurs mainly in series resulting in longitudinal cell growth. In the transition to maladaptive LVH, LV dilatation becomes disproportional to wall thickness, with myocytes elongated without an increase in diameter.

Experimental models of cardiac hypertrophy support the theory that mechanical stress due to either pressure or volume overload is a trigger for activation of other multiple mechanisms leading to myocardial remodeling [62]. These factors include a local overexpression of the renin-angiotensin-aldosterone system (RAAS), adrenergic system, inflammatory cytokines and other autocrine and paracrine mechanisms. In patients with CKD, these mechanisms might be activated independently of hemodynamic overload since uremia per se is associated with an alteration in multiple humoral factors [63, 64]. With time, a maladaptive phase of LVH develops, characterized by decreased capillary density, decreased coronary reserve and subendocardial perfusion, a tendency to arrhythmia, and the development of myocardial fibrosis. All this leads to myocyte death and, finally, to diastolic and systolic dysfunction.

The second process involves vascular injury. Exposure to CV risk factors results in vascular changes, including atherosclerotic and arteriosclerotic processes and vascular calcification.

Atherosclerosis refers to the process of plaque formation or atheroma development. The process of atheroma formation begins with an accumulation of lipid-containing foam cells (macrophages) in the vascular intima and evolves into successive structures that penetrate the vascular wall and include lipids, smooth muscle cells and collagen fibers [65]. Calcification is an intrinsic part of the process and generally involves the intima. Atherosclerotic lesions have a patchy distribution along the length of the artery and cause local stenoses and occlusions. Recently, endothelial progenitor cells (EPCs) have been identified as contributing to angiogenesis [66]. The EPC number has been shown to be reduced in patients with CVD, leading to speculation that atherosclerosis may be caused by a consumptive loss of the endothelial repair capacity. Animal experiments have shown that EPCs reendothelialise injured vessels and that this reduces neointimal formation, confirming that EPCs have an atheroprotective effect.

Arteriosclerosis is arterial stiffening involving the entire arterial tree, although it principally affects the elastic arteries. Unlike atherosclerosis, arteriosclerosis involves both intimal and medial thickening. In CKD, arteriosclerosis can occur in the absence of significant atherosclerotic disease [67–69]. Arteriosclerosis is associated with vascular remodeling characterized by increased wall thickness, lumen enlargement and increased length of arteries. This leads to an increase of systolic BP and pulse pressure and arterial stiffening.

The triggers for vascular calcification are complex and include metabolic, mechanical, infectious and inflammatory injuries. Increased calcium x phosphate ion product or hyperphosphatemia may be the key promoter of vascular calcification [70]. The mechanisms include either stimulation of the uptake and precipitation of calcium and phosphate into the vessel or a decrease of the inhibitory process that prevents these ions from precipitation. Fetuin-A, the anti-inflammatory protein, is a highly potent inhibitor of serum calcium-phosphate complex formation [71, 72]. The serum concentrations of Fetuin-A are decreased in ESRD patients [73]. Another promoter of vascular calcification is 1,25(OH)_2_D_3_, which may have a direct effect on the calcium deposition in vascular smooth muscle cells [74]. In a comprehensive review, Vattikuti and Towler [75] defined four major histo-anatomic variants of vascular calcification, including classic atherosclerotic (fibrotic) calcification related to disorders of lipid metabolism, medial arterial calcification, vascular calcifilaxis and cardiac valve calcification.

## Spectrum of cardiovascular abnormalities in children with CKD

Over the last decade, abnormalities of the LV such as LVH and LV dysfunction, abnormalities of the large arteries such as stiffness and increased intima-medial thickness (IMT) of the carotids, and calcification of the coronaries have been accepted as early markers of cardiomyopathy and atherosclerosis. They constitute strong independent predictors of cardiac morbidity and mortality both in the general population and in adults with CKD. In children and young adults with CKD, recent studies have proven that these abnormalities are also present and that risk factors for cardiac and vascular injury in children with CKD are similar to those for adults.

## Left ventricular hypertrophy

LVH develops when renal insufficiency, is mild or moderate in children and progresses as renal function deteriorates. About one third of children with mild to moderate renal insufficiency have an increased left ventricular mass (LVM) index [76–78]. In a 2-year prospective longitudinal study of 31 pediatric subjects with CKD stage 2–4, Mitsnefes et al. [79] showed that a substantial proportion of children had a significant increase in the LVM index, with many of the children developing LVH. Indeed, 32% of the patients who initially had a normal LVM index have developed incident LVH. At initiation of maintenance dialysis, 69–82% of pediatric patients have evidence of LVH [80, 81]. LVH persists (40–75%) during long-term dialysis [8, 76, 82–85], with both concentric and eccentric geometric patterns of LVH present in these patients. Post-mortem studies confirmed high rate (more than 50%) of LVH in children with ESRD [86]. Small retrospective studies also suggest that with a better BP and volume control, LVH regression might be achieved in young patients on dialysis [80, 81]. On the other hand, a recent retrospective study demonstrated that LVH remains very prevalent and severe in a selected group of children who remained on maintenance dialysis for at least 2 years [9].

As in children prior to transplantation, most pediatric studies indicate that LVH remains common post transplant (48–82%) [87–91]. In contrast, a significantly lower frequency of LVH was found in a study by Englund et al. [92], who reported the results of a longitudinal analysis of children receiving renal transplants 10–20 years ago. Of 53 children who received a renal transplant between 1981 and 1991, 47 survived and were observed for 10 to 20 years. At the 10-year follow-up, echocardiography showed minor LVH in only two children with hypertension. No child without hypertension at 10 years post transplant had LVH.

The factors associated with cardiac hypertrophy in children are similar to those in adults with CKD. As in adults, most pediatric studies of patients with pre-terminal, terminal renal failure and post transplant found significant relationships between low hemoglobin and an increased LVM index [8, 78, 79, 90]. However, recent adult studies in mild to moderate CKD or in chronic dialysis determined that correction of anemia was not associated with regression of LVH [93–96]. Authors suggested that relationships between anemia and LVH might not be causal. Of note, the above studies enrolled subjects with relatively mild degrees of baseline anemia and could not answer the question whether treatment of patients with initially significantly decreased hemoglobin levels might lead to a reduction of LVM. In contrast, Morris et al. [97] observed a significant reduction in the LVM index with the correction of severe anemia in seven children on chronic dialysis.

There are several studies on the association between parathyroid hormone (PTH) levels and LVH in adults with CKD [98, 99]. In children, elevated PTH is associated with progression of LVH in stages 2–4 CKD [79]. The possible mechanisms of parathyroid-induced cardiac hypertrophy in CKD include a direct effect of PTH on cardiomyocytes and an indirect effect via elevated BP [99]. A support for a causal relationship comes from in vitro studies showing that PTH appears to have chronotropic, inotropic as well as hypertrophic effects on cardiomyocytes [100, 101].

In adults with CKD, hypertension is directly linked to the development of LVH [39]. The relationships between BP and LVH in pediatric CKD are unclear. Consistent correlations of LVM and BP are limited to children with ESRD [8, 85]. However, a detailed cross-sectional analysis of BP characteristics by ambulatory blood pressure monitoring (ABPM) in children from the ESCAPE trial did not demonstrate any relationship between office BP or ABPM parameters and LVM, suggesting only a minor role of hypertension in the pathogenesis of LVH in early CRI [78]. In contrast, analysis of longitudinal data suggests that ABPM might be an important tool to assess the risk of development of LVH in children with CKD [79]. In this study, authors determined that an increase in the nighttime systolic BP load (number of BP measurements above the 95th percentile BP value) were independently associated with the increase in the LVM index over time, arguing that persistent and chronic elevation of BP might be more important in the development of LVH [79].

## Left ventricular function

In contrast to adults, in whom systolic dysfunction is frequently associated with early cardiac failure and decreased survival, in children with CKD systolic LV function is usually preserved [76, 102–104]. On the other hand, diastolic dysfunction, often the initial abnormality of cardiac function, is already present in children with CKD. Doppler measurement of mitral inflow velocity has been the most widely used method to assess LV diastolic function. Using this method, Goren et al. [103] showed that LV relaxation (E/A ratio) was impaired in dialyzed children as compared to controls. Johnstone et al. [76] also found a reduction in the E/A ratio in children on chronic peritoneal dialysis and with pre-terminal renal failure, although none of these patients had an E/A ratio <1.0, which is considered to be abnormal. Unfortunately, the transmitral Doppler velocities and, therefore, the E/A ratio, are affected by several factors, including left atrial pressure and preload. This is particularly important for patients with advanced chronic renal failure, since many of them are hypervolemic. Recently, new indices were introduced to evaluate diastolic function using tissue Doppler imaging (TDI). In contrast to E/A, the TDI indices may be less load dependent and provide a more accurate measure of diastolic function. Recent studies employing TDI determined that children with CKD might have abnormal diastolic function [105, 106]. In these studies, children on chronic dialysis had significantly worse diastolic dysfunction than children with mild-to-moderate CRI or post transplant. Poor diastolic function in patients on dialysis was associated with anemia, hyperphosphatemia, increased calcium-phosphorus ion product and LVH. The clinical significance of diastolic dysfunction in pediatric patients with CKD is not known. Longitudinal studies are necessary to determine if abnormal diastolic function predicts the development of systolic dysfunction and congestive heart failure in these patients.

## Arterial structure and compliance

Studies of young adults who developed ESRD during childhood found a high prevalence of abnormal carotid IMT, diminished arterial wall compliance and coronary artery calcification (CAC). These vascular abnormalities are accepted as markers of asymptomatic atherosclerosis and predictors of future symptomatic CVD in the general population and in adults with CKD [107, 108]. Groothoff et al. [109] found increased arterial stiffness and showed that systolic hypertension was the main determinant of abnormal arterial wall compliance. Goodman et al. [110] showed that among 23 patients on chronic dialysis who were younger than 20 years of age, none had evidence of CAC; in contrast, 14 of the 16 patients who were 20 to 30 years of age had evidence of CAC on CT scanning. Oh et al. [3] screened for coronary and carotid artery disease in 39 patients, aged 19 to 39 years, with childhood onset ESRD. Coronary artery calcification was present in 92% and carotid IMT was significantly increased compared to matched controls. Carotid IMT was correlated with cumulative dialysis and serum Ca x P product in their study. Milliner et al. [111], in an autopsy study of pediatric patients with ESRD who died in 1960–1983, showed a high prevalence of soft tissue and vascular calcinosis. In their study, CAC was present in 28%. Peak Ca x P product, peak serum P and cumulative dose of calcitriol were significantly associated with the severity of the calcinosis. Civilibal et al. [112] screened 53 children with ESRD for the presence and predisposing factors of CAC. Coronary artery calcification was present in 15% of patients (three hemodialysis patients, three peritoneal dialysis patients and two renal transplant recipients). The patients with CAC had a longer duration of total dialysis, had higher time-integrated serum phosphorus, calcium-phosphate product, intact parathyroid hormone, vitamin B12 levels and the amount of cumulative calcium-containing oral phosphate binders. In a study by Briese et al. [113] of 40 young adults (mean age 23.6 years) who developed ESRD at a mean age of 11.5 years, carotid artery IMT was similar to healthy controls and only 4 (10%) patients had evidence of coronary calcification. Authors noticed that a relatively low rate of cardiac calcification compared to other studies might be explained by a significantly lower amount of prescribed calcium-containing phosphate binders and vitamin D preparations in their patients. Of note, these patients had decreased vascular reactivity and a high rate of LVH (68.2%). As in adults, cardiac valve calcification was also described in children on maintenance dialysis [114].

Evidence of early atherosclerotic changes was found in the study by Nayir et al. [115], who reported on the histopathology of internal iliac artery samples obtained at the time of kidney transplantation in 12 children. Five arteries had fibrous or fibroelastic intimal thickening, medial mucoid ground substance and disruption of the internal elastic lamella. Two of these had microcalcification in the intimal layer; another two demonstrated atheromatous plaques. These abnormalities were associated with longer duration of renal failure.

Vascular abnormalities in children develop in parallel with cardiac abnormalities early in the course of CKD and become more severe as end-stage disease is reached [116]. Mitsnefes et al. [54] showed that carotid arteriopathy is present in children after successful renal transplantation and is associated with hypertension. Litwin et al. [117], as a part of the ESCAPE trial, investigated vascular structure and function in children with chronic renal failure and after renal transplantation. The authors demonstrated vascular abnormalities in all patient groups with the most marked changes in the dialysis patients. The degree of arteriopathy in their study was correlated with conventional CVD risk factors such as hypertension and dyslipidemia in pre-dialytic CKD, while in children on dialysis and after transplantation, hyperphosphatemia, hyperparathyroidism and treatment with calcium-containing phosphate binders were determinants of arterial abnormalities. Another important observation in this study was a significantly lower carotid IMT in children post transplant, suggesting that the vascular abnormalities partially regress. Epidemiological studies should determine if vascular abnormalities detected during childhood CKD are associated with future accelerated coronary artery disease.

## Endothelial dysfunction as a marker of early atherosclerosis in children with CKD

Endothelial function can be evaluated by the assessing of endothelial vasodilatation. Healthy vascular endothelium will respond to the release of nitric oxide by vasodilatation. With endothelial injury, the response to nitric oxide is diminished [118], and endothelial-mediated vasodilatation is impaired. Flow-mediated dilation (FMD) of the brachial artery is currently used to measure endothelial function. This test consists of applying obstruction to the flow of the brachial artery by placing the tourniquet or inflated BP cuff for few minutes. The artery dilates due to post-obstruction reactive hyperemia. FMD is calculated based on the difference in the diameter of the brachial artery before obstruction and after it released. Impaired (decreased) FMD predicts CVD morbidity and mortality in adults with ESRD [119]. It has been shown that endothelial dysfunction as determined by impaired FMD is also present in children with advanced renal failure, on chronic dialysis and after renal transplantation [120–122].

## Evaluation and treatment recommendations

The overall strategy in the prevention of cardiovascular complications in children with CKD is avoidance of long-term dialysis. The goal is to prevent the development and delay the progression of cardiomyopathy and atherosclerosis. The identification of modifiable risk factors and markers of CVD and early intervention should be initiated at the time of mild-to-moderate renal insufficiency, prior to the need for dialysis. Even though kidney transplantation poses a continuous CV risk (hypertension, hyperlipidemia and allograft dysfunction), it eliminates many uremia-related risks, reduces the risk of cardiac death by approximately 80 percent and prolongs the life span by 20–30 years. Thus, kidney transplantation should be the ultimate goal to minimize cardiovascular morbidity and mortality in patients with advanced CKD. For those patients who must have long-term dialysis, the strategy is directly linked to achievement of optimal dialysis outcomes, which include aggressive monitoring and management of hypertension, dyslipidemia, calcium-phosphorus metabolism, anemia, nutrition, systemic inflammation and other dialysis complications.

Current recommendations on evaluation of CV abnormalities and treatment of modifiable risk factors in children are based mostly on clinical experience and adult data. Recent K/DOQI Clinical Practice Guidelines for Cardiovascular Disease in Dialysis Patients [42] recommend echocardiographic evaluation for the presence of cardiac disease in children (cardiomyopathy and valvular disease) at the time of initiation of dialysis therapy along with the screening for dyslipidemia, hypertension, anemia and increased Ca×P product. Management of modifiable risks in children with CKD should follow recommendations from the K/DOQI guidelines for treatment of anemia [123] and dyslipidemia [15] and from the Fourth Report on Blood Pressure in Children for Management of Hypertension [124]. Current K/DOQI guidelines for treatment of anemia recommend keeping the hemoglobin level above 11 g/dl by using an appropriate iron therapy and recombinant erythropoietin. In the opinion of the Working Group, there is insufficient evidence to recommend routinely maintaining hemoglobin levels above 13 g/dl or more.

K/DOQI recommends evaluation of dyslipidemia in adolescents upon presentation with CKD stage 5 (GFR <15 ml/min/1.73 m^2^ or on dialysis), at 2–3 months after a change in treatment or other conditions known to cause dyslipidemia and at least annually thereafter. Reasons to repeat lipid measurements after 2–3 months include changes in the kidney replacement therapy modality, treatment with diet or lipid-lowering agents, immunosuppressive agents that affect lipids (e.g., prednisone, cyclosporine or sirolimus) or other changes that may affect plasma lipids. The assessment of dyslipidemia should include a complete fasting lipid profile with total cholesterol, LDL, HDL and triglycerides. The definition of dyslipidemia differs in children and adults. Hyperlipidemia in children is defined as lipid levels greater than the 95th percentile for age and gender. The normative data for lipids in children and adolescents currently used are from the Lipid Research Clinics Program from the NIH published in 1980 and can be found in the 2003 K/DOQI guidelines for management of dyslipidemia in chronic kidney disease [15].

For adolescents with stage 5 CKD and a level of LDL ≥130 mg/dl, K/DOQI recommends treatment to reduce LDL to <130 mg/dl. If LDL is <130 mg/dl, fasting triglycerides ≥200 mg/dl and non-HDL cholesterol (total cholesterol minus HDL) ≥160 mg/dl, treatment should be considered to reduce non-HDL cholesterol to <160 mg/dl. All children with dyslipidemia should follow the recommendations for therapeutic lifestyle changes (TLC), which include diet modification with a reduction in saturated fat intake and increase in fiber intake, and moderate physical activity. Adolescents should be counseled about avoiding smoking. Unfortunately, non-compliance with TLC is one of the major problems in the management of dyslipidemia in adolescents. Pediatric nephrologists must also recognize that appropriate caloric intake, including calories from fat, should be emphasized to avoid malnutrition and ensure normal growth and development, especially in young children. If LDL cholesterol is ≥160 mg/dl and non-HDL cholesterol ≥190 mg/dl, statin therapy is recommended in children older than 10 years.

Target blood pressure in children should be lower than the 90th percentile for normal values adjusted for age, gender and height or less than 120/80 mm Hg, whichever is lower. Ambulatory blood pressure monitoring is recommended to assess the circadian rhythm. Angiotensin-converting enzyme inhibitors or angiotensin receptor blockers may be the preferred antihypertensive agents to slow the progression of CKD in children and possibly for regression of LVH. The Working Group on Cardiovascular Disease in Dialysis Patients recommends maintaining calcium and phosphorus levels within the normal range and the Ca×P product <55 mg^2^/dl^2^ in children on chronic dialysis [42].

There are no data available to make evidence-based recommendations on management of hyperhomocysteinemia, chronic inflammation or other potential CVD risk factors.

**Questions**(Answers appear following the references)

1. A 14-year-old African-American boy with ESRD secondary to FSGS has been treated with thrice-weekly hemodialysis for 1 year. His pre-dialysis BP is 132/85, hemoglobin is 9.8 g/dl, serum calcium is 10.6 g/dl, serum phosphorus is 8.2 g/dl and iPTH is 412 pg/ml. He is taking a calcium channel blocker to control his hypertension, erythropoietin and iron to control his anemia and calcium carbonate and a vitamin D IV preparation to control his renal bone disease.
  **Which ONE of the following statements would be the BEST therapeutic intervention in this child to minimize the risk of future CV complications?**
  - Aggressive treatment of hypertension
  - Maximize dialysis treatment to achieve dry weight
  - Correction of anemia
  - Kidney transplantation
  - Reduction of Ca-P product
2. The patient from the previous question was diagnosed with eccentric LVH.
  **With regard to treatment of his LVH, which ONE of the following actions is MOST LIKELY to lead to reduction of LVH?**
  - Adding ACE inhibitor as a second antihypertensive agent
  - Increase the dose of erythropoietin and assure appropriate iron status to treat anemia
  - Achievement of dry weight
  - Switch from hemodialysis to peritoneal dialysis
  - Maximize treatment of secondary hyperparathyroidism
3. The annual evaluation showed that this patient has LDL cholesterol of 170 mg/dl. You would like to start this patient on atorvastatin.
  **With regard to the use of “statin” in this patient, which ONE of the following statements is correct?**
  - Statins are not approved by the United States Food and Drug Administration (USFDA) for use in children and adolescents
  - Grapefruit is contraindicated
  - The dose of atorvastatin should be reduced by 50% compared to dosage in adult patients on dialysis to prevent adverse effects on growth and development
  - The target level for LDL cholesterol in children with CKD should be below 100 mg/dl
  - Atorvastatin therapy will reduce mortality risk in this patient
4. **Which ONE of the following statements concerning development of cardiovascular complications in a child with CKD is NOT correct?**
  - Cardiovascular disease is the cause of mortality in approximately 25% of chronically dialyzed children
  - Cardiac arrest is the most common cardiac cause of death in children with ESRD
  - Cardiac and vascular remodeling might develop in children during early stages of CKD
  - Correction of anemia reduces concentric LVH
  - LVH is the most common cardiac abnormality in children on maintenance dialysis
5. The patient described in the previous questions is at risk for development of vascular calcification.
  **Which ONE of the following choices does NOT correctly describe the mechanisms of vascular calcification in dialyzed patients?**
  - Histo-anatomic variants of calcification include classic atherosclerotic (fibrotic) calcification related to disorders of lipid metabolism, medial arterial calcification, vascular calcifilaxis and cardiac valve calcification
  - Increased Ca-P product and hyperphosphatemia are the key drivers of vascular calcification in young patients with CKD
  - Inflammatory mechanisms are involved in mediating all stages of atherosclerosis including calcification
  - Increased serum level of Fetuin-A promotes Ca-P ion product precipitation
  - 1, 25 (OH)_2_D_3_ increases calcium deposition in the vascular wall

Answers

1. d
2. c
3. b
4. d
5. d
